# Nicotinamide Improves Cognitive Function in Mice With Chronic Cerebral Hypoperfusion

**DOI:** 10.3389/fneur.2021.596641

**Published:** 2021-01-25

**Authors:** Bin Liu, Guifeng Zhao, Ling Jin, Jingping Shi

**Affiliations:** ^1^Department of Geriatrics, The Affiliated Brain Hospital of Nanjing Medical University, Nanjing Brain Hospital, Nanjing, China; ^2^Key Laboratory of Research and Application of Animal Models for Environmental and Metabolic Diseases, Sheng Jing Hospital of China Medical University, Shenyang, China; ^3^Department of Neurology, The Affiliated Brain Hospital of Nanjing Medical University, Nanjing Brain Hospital, Nanjing, China

**Keywords:** chronic cerebral hypoperfusion, cognitive impairment, memory, nicotinamide, white matter

## Abstract

Normal brain function requires steady blood supply to maintain stable energy state. When blood supply to the brain becomes suboptimal for a long period of time, chronic cerebral hypoperfusion (CCH) and a variety of brain changes may occur. CCH causes white matter injury and cognitive impairment. The present study investigated the effect of nicotinamide (NAM) on CCH-induced cognitive impairment and white matter damage in mice. Male C57Bl/6J mice aged 10–12 weeks (mean age = 11 ± 1 weeks) and weighing 24 - 29 g (mean weight = 26.5 ± 2.5 g) were randomly assigned to three groups (eight mice/group): sham group, CCH group and NAM group. Chronic cerebral hypoperfusion (CCH) was induced using standard methods. The treatment group mice received intraperitoneal injection of NAM at a dose of 200 mg/kg body weight (bwt) daily for 30 days. Learning, memory, anxiety, and depression-like behaviors were measured using Morris water maze test (MWMT), open field test (OFT), sucrose preference test (SPT), and forced swim test (FST), respectively. White matter damage and remodeling were determined via histological/ immunohistochemical analyses, and western blotting, respectively. The results showed that the time spent in target quadrant, number of crossings and escape latency were significantly lower in CCH group than in sham group, but they were significantly increased by NAM (*p* < 0.05). Mice in NAM group moved significantly faster and covered longer distances, when compared with those in CCH group (*p* < 0.05). The percentage of time spent in open arms and the number of entries to the open arms were significantly lower in CCH group than in NAM group (*p* < 0.05). Moreover, anhedonia and histologic scores (index of myelin injury) were significantly higher in CCH group than in sham group, but they were significantly reduced by NAM (*p* < 0.05). The results of immunohistochemical staining and Western blotting showed that the protein expressions of 2′, 3′-cyclic-nucleotide 3′-phosphodiesterase (CNPase) and synaptophysin were significantly downregulated in CCH group, relative to sham group, but they were significantly upregulated by NAM (*p* < 0.05). These results indicate that NAM improves cognitive function in mice with CCH.

**Graphical Abstract:**
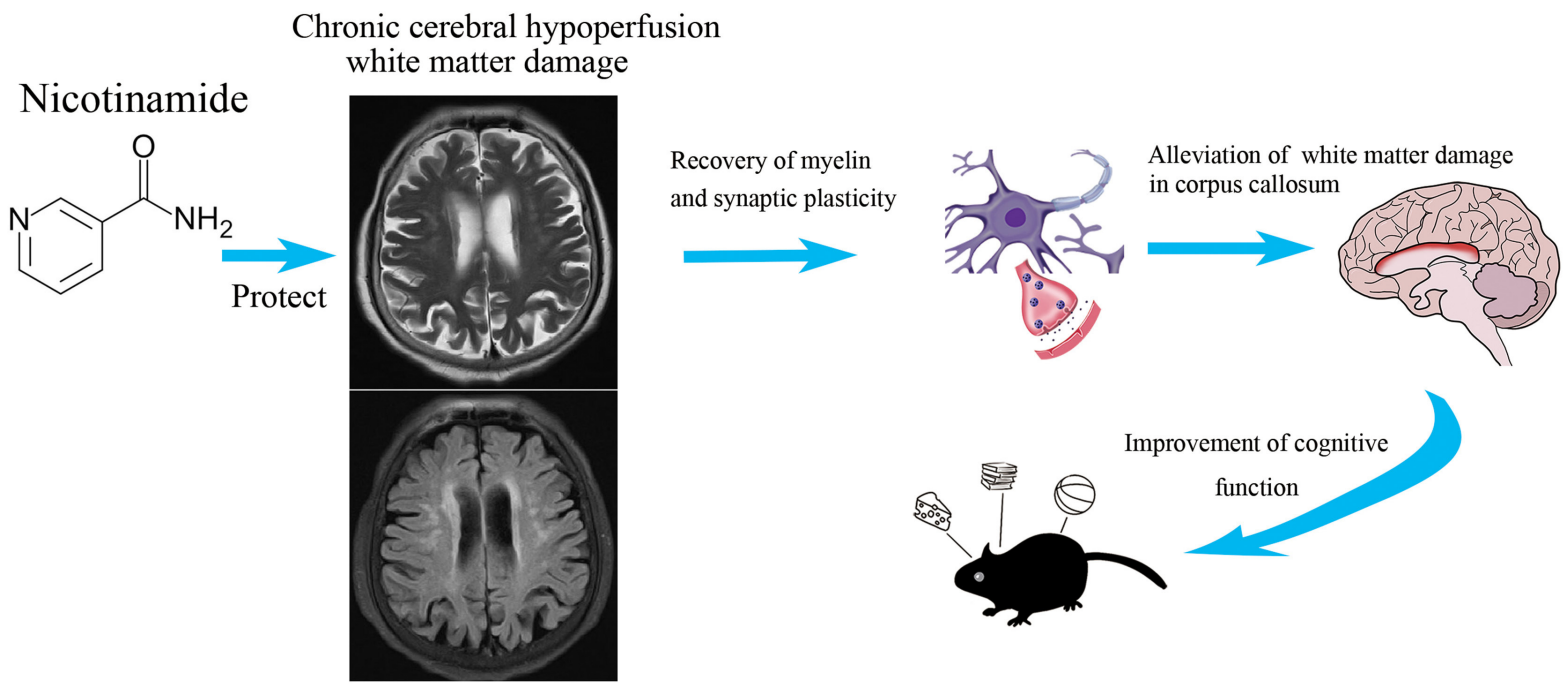
Nicotinamide has extensive neuroprotective effects on cognitive impairment after chronic cerebral ischemia.

## Introduction

Chronic cerebral hypoperfusion (CCH)-induced white matter lesions are usually seen in subcortical and periventricular regions of the brain during magnetic resonance imaging (MRI) and computed tomography (CT) scans. Subcortical MRI lesions are common in the elderly: it occurs in 30–92% of patients older than 60 years of age and also in 22% of persons under the age of 40. The lesions are found scattered throughout the deep cerebral white matter, notably in the frontal and parieto-occipital periventricular regions, and basal ganglia, especially the globus pallidus and putamen (1).

Studies have shown that CCH-induced white matter lesions contribute to recurrent stroke, Alzheimer's disease, as well as Parkinson disease (2, 3). At present there is no effective treatment for CCH-induced white matter damage. White matter damage, especially in the corpus callosum adjacent to the lateral ventricle, is associated with impaired memory and balance. Cognitive impairment is the hallmark of CCH-induced white matter injury. In mouse model of CCH, changes in white matter have been shown to be closely associated with cognitive deficits. Cognitive impairment is usually measured using MWMT, OFT, and FST (4, 5).

Oligodendrocytes and synapses are important components of white matter, and they contribute to neuronal connectivity and function in adult brain. Autopsies of patients with white matter lesions caused by small vessel diseases revealed myelin damage and degeneration of synapses. These lesions are characterized by marked reduction in oligodendrocytes and degenerated synaptic junctions (6, 7). Therefore, there is need for strategies that can effectively alleviate white matter damage and ultimately reduce neurological deficits induced by CCH.

Breakdown of the blood–brain barrier and increased permeability, oxidative stress, and inflammation have been cited as possible important causes in the development of white matter changes in CCH (8–10). The series of damage process is slow and moderate, compared with acute cerebral ischemia. However, whether the drugs for acute cerebral ischemia are suitable for white matter injury after chronic hypoperfusion remains to be studied. Early use of drugs might improve cognitive impairment, and the time point, dose and duration of drug administration also need to be further discussed.

Nicotinamide (NAM), an essential precursor of nicotinamide adenine dinucleotide (NAD^+^), inhibits apoptosis via regulation of oxidative stress. This molecule has shown great promise in the treatment of chronic diseases such as type 1 diabetes mellitus, Friedreich's ataxia, skin cancer, Parkinson's disease and Huntington's disease (11, 12). Derivatives of NAM could improve the damage of blood-brain barrier, reduce inflammatory reaction, and promote angiogenesis (13–15). Moreover, in focal ischemia-induced brain injury, long-term administration of NAM confers some neuroprotection. Studies have demonstrated that NAM reduces infarct volume, promotes myelination and regulates the expression levels of several proteins (11, 16, 17). The protein pathways involved include PARP against apoptosis, CD38 for the activity of this NADase, the NAD-dependent deacetylase SIRT1, BDNF as neurotrophin, activating protein kinase B for the regulation of inflammation (18–21). The present study investigated the effect of NAM on CCH-induced cognitive impairment and white matter damage in mice.

## Materials and Methods

### Materials

Rabbit anti-CNPase and synaptophysin polyclonal antibodies were obtained from Bioss (China) and ProteinTech (China), respectively. β-Actin and horseradish peroxidase (HRP)-labeled goat anti-rabbit IgG were purchased from Cell Signaling Technology Co. Ltd (USA). Bicinchoninic acid (BCA) protein assay kit and 3, 3′ diaminobenzidine (DAB) were products of Thermo Fisher Scientific Co (USA). Image-Pro Plus 6.0 was purchased from Media Cybernetics, Inc (USA). Image analysis system was bought from Imaging Research (Canada).

### Experimental Mice

Male C57Bl/6J mice aged 10–12 weeks (mean age = 11 ± 1 weeks) and weighing 24–29 g (mean weight = 26.5 ± 2.5 g) were used for this study. The mice were housed in metal cages under standard conditions and allowed free access to standard feed and water. Prior to commencement of study, the mice were acclimatized to the laboratory environment for 3 days. Thereafter, they were exposed to 12-h light/12-h dark cycle, and maintained at an average temperature of 24 ± 3°C, and 55–65% humidity. The study protocol was approved by the Institutional Animal Care and Use Committee of Nanjing Medical University (No. IACUC-2007034). The procedures used were carried out according to the guidelines of the Association for the Assessment and Accreditation of Laboratory Animal Care International (AAALAC).

### Experimental Design

**Figure 1:**
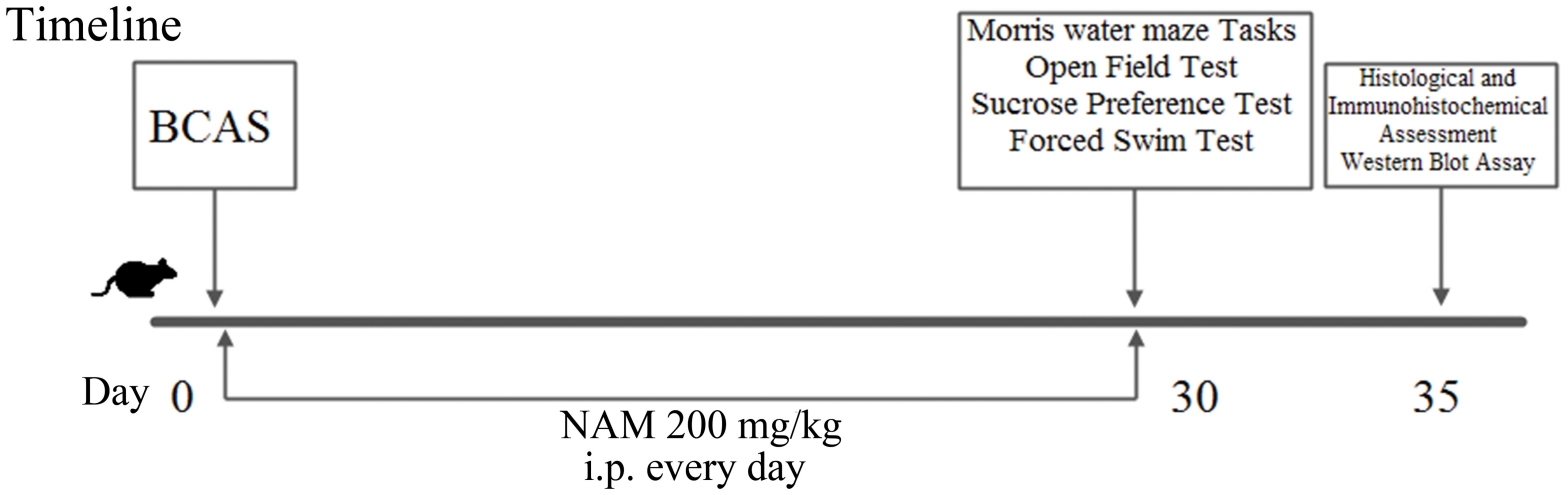
Scheme showing the experimental design of the study.

The mice (*n* **=** 24) were randomly assigned to three groups (eight mice/group): sham group, CCH group and NAM group. Chronic cerebral hypoperfusion (CCH) was induced using standard methods (8). The mice were anesthetized with 1.5% isoflurane. Incision was made on the neck of each mouse to expose the common carotid artery (induction of bilateral common carotid artery stenosis, BCAS). Microcoil (inner diameter = 0.18 mm) was then wrapped around the common carotid artery. Sham operated mice were exposed to the same surgical procedures as CCH mice, but without implantation of microcoils. Body temperature of each mouse was maintained at 37 ± 0.5°C. Mice in the treatment group received a single daily dose of NAM (200 mg/kg bwt) intraperitoneally (i.p.) for 30 days (17). Treatment was initiated 2 h after the onset of BCAS. All mice were used in behavioral analysis. After neurological function tests, four mice in each group were used for histopathological examination and immunohistochemical staining, the other four mice in each group were subjected to Western blot assay. The experimental design is shown in Figure 1.

### Measurement of Neurological Function in CCH Mice

Behavioral analysis (*n* = 8/group) was carried out using MWMT, OFT and sucrose preference test (SPT) and forced swim test (FST).

### Morris Water Maze Test

Spatial learning was measured using MWMT. The apparatus consisted of a circular water pool (45 cm in height and 120 cm in diameter), with a smooth inner surface. The water-maze tank was placed with external visual cues and filled with water. The temperature of the water was maintained at 25 ± 2°C. In one of the equally divided quadrants, a platform (30 cm in height and 10 cm in diameter) was submerged 2 cm in the water. The time spent to find the platform (escape latency) was recorded during each acquisition trial session. A 4-day memory acquisition training included two trial sessions at intervals of 3–4 h per day. After completion of acquisition training, MWMT was conducted on days 6, 10, and 14 of treatment. On location of the platform, a mouse was allowed to stay on it for 10 s. Any mouse that failed to locate the platform within 2 min was guided to it and allowed to stay on it for another 10 s. On the last day, a probe trial was conducted by removing the hidden platform from the water pool. Mice were allowed to swim and the time spent in the target quadrant where the platform was previously located was recorded (22). The data were analyzed using Ethovision 13 system.

### Open Field Test (Locomotor Activity Assay)

Each mouse was placed in an open-field arena (50 × 50 × 40 cm) and allowed free exploration within 30 min. Video tracking was used to record horizontal movement of mice over six consecutive blocks of 5 min each. The total distance traveled in the central zone (30 × 30 cm square field of the appliance), and the time spent in the center were recorded within 120 min and analyzed using Ethovision 13 system (22).

### Sucrose Preference Test

Sucrose preference test (SPT) is a measure of the ability to experience pleasure as a feature of clinical depression (anhedonia). Anhedonia is the inability to experience pleasure from rewarding or enjoyable activities, and it is a core symptom of depression in humans (22). Mice were made to choose between 1% sucrose solution and tap water for 48 h. Then, their preference for one over the other was measured within 24 h. Sucrose preference was calculated as shown in Equation (1):

(1) $$\begin{array}{l} Sucrose\;preference=\left(sucrose\;consumption\right)\times 100\ldots \ldots \ldots \\ \;\left(sucrose\;consumption+water\;consumption\right) \end{array}$$

### Forced Swim Test

Forced swim test (FST) produces a behavioral situation in mice similar to depression in humans (hopelessness manifested in a period of immobility). In FST, an open miniature swimming pool of dimensions 25 × 15 × 25 cm maintained at a temperature of 25 ± 2°C was used. One hour after the 14th day treatment, mice were dropped down in water and their activities were recorded within 4 min after an initial 2 min of vigorous escape attempt (22).

### Histopathological Examination of Mice Brain Tissues

Excised brain tissues (*n* = 4/group) were fixed via transcardial perfusion with saline, followed by immersion in 4% paraformaldehyde. The tissues were thereafter embedded in paraffin. Serial sections (3 μm thickness) were made using a microtome and stained with hematoxylin and eosin (H&E) and Klüver-Barrera according to standard method, and examined under light microscope. Image-Pro Plus, 6.0 (IPP, 6.0) was used to determine histopathological changes in white matter fibers and vacuoles (vacuole-like holes in the white matter). The percentage of area of vacuole-like holes in corpus callosum were counted according to IPP, 6.0. The severity of white matter rarefaction was graded according to standard grading system: 0 = normal; 1 = disorganization of nerve fibers; 2 = formation of marked vacuoles; and 3 = disappearance of myelinated fibers (23).

### Immunohistochemical Staining

Brain tissue sections (3-μm thickness) were subjected to immunohistochemistry (IHC). The sections (*n* = 4/group) were deparaffinized, rehydrated, and incubated in 3% H_2_O_2_ for 10 min to reduce non-specific background staining. Then, the tissue samples were agitated for 15 min in 10 mM citrate buffer (pH 6.0) in a microwave oven. The tissue sections were thereafter incubated for 10 min at room temperature in Ultra V Block solution. This was followed by the addition of rabbit anti-CNPase and synaptophysin polyclonal antibodies (1:250), and incubation for 2 h at room temperature. Antibody binding was determined using Ultra-vision LP System according to the manufacturer's instructions. The sections were developed using DAB, and counterstained with hematoxylin. The expressions of 2′, 3′-cyclic-nucleotide 3′-phosphodiesterase (CNPase) and synaptophysin were scored. Zero (0) score was considered negative for CNPase and synaptophysin, while scores of 1+ to 4+ were taken for over-expressions of CNPase and synaptophysin (24). The area of the expression of protein and the corpus callosum were counted according to IPP, 6.0. The percentage of protein expression area in corpus callosum was compared in different groups.

### Western Blotting

Cell suspension (*n* = 4/group) resulting from trypsinization of brain tissues from the corpus callosum was washed twice with phosphate-buffered saline (PBS) and lysed with NP40 protein lysis buffer containing protease and phosphatase inhibitors at a volume ratio of 1:5. The resultant lysate was centrifuged at 12,000 rpm for 10 min at 4°C, and the protein concentration of the supernatant was determined using BCA protein assay kit. A portion of total cell protein (30 μg) from each sample was separated on a 10% sodium dodecyl sulfate (SDS)-polyacrylamide gel electrophoresis and transferred to a fixed polyvinylidene fluoride membrane at 110 V and 90°C for 120 min. Subsequently, non-fat milk powder (5%) in Tris-buffered saline containing 0.2% Tween-20 (TBS-T) was added to the membrane, with gentle shaking at 37°C and incubated to block non-specific binding of the blot. Thereafter, the blots were incubated overnight at 4°C with primary antibodies (rabbit polyclonal anti-CNPase, anti-synaptophysin and anti-β-actin, each at a dilution of 1 to 1,000). Thereafter, the membrane was washed thrice with TBS-T and further incubated with horseradish peroxidase-conjugated goat anti-rabbit IgG secondary antibody for 2 h at room temperature. The blot was developed using an X-ray film. Grayscale analysis of the bands was performed using enhanced chemiluminescence (ECL). The various protein expression levels were normalized to that of β-actin (24).

## Statistical Analysis

Data are expressed as mean ± SD. Statistical analysis was performed using GraphPad prism (8.0). Data were analyzed with two-way ANOVA followed by Bonferroni *post hoc* test. Statistical significance was assumed at *p* < 0.05.

## Results

### Effect of NAM on Mouse Cognitive Function

**Figure 2:**
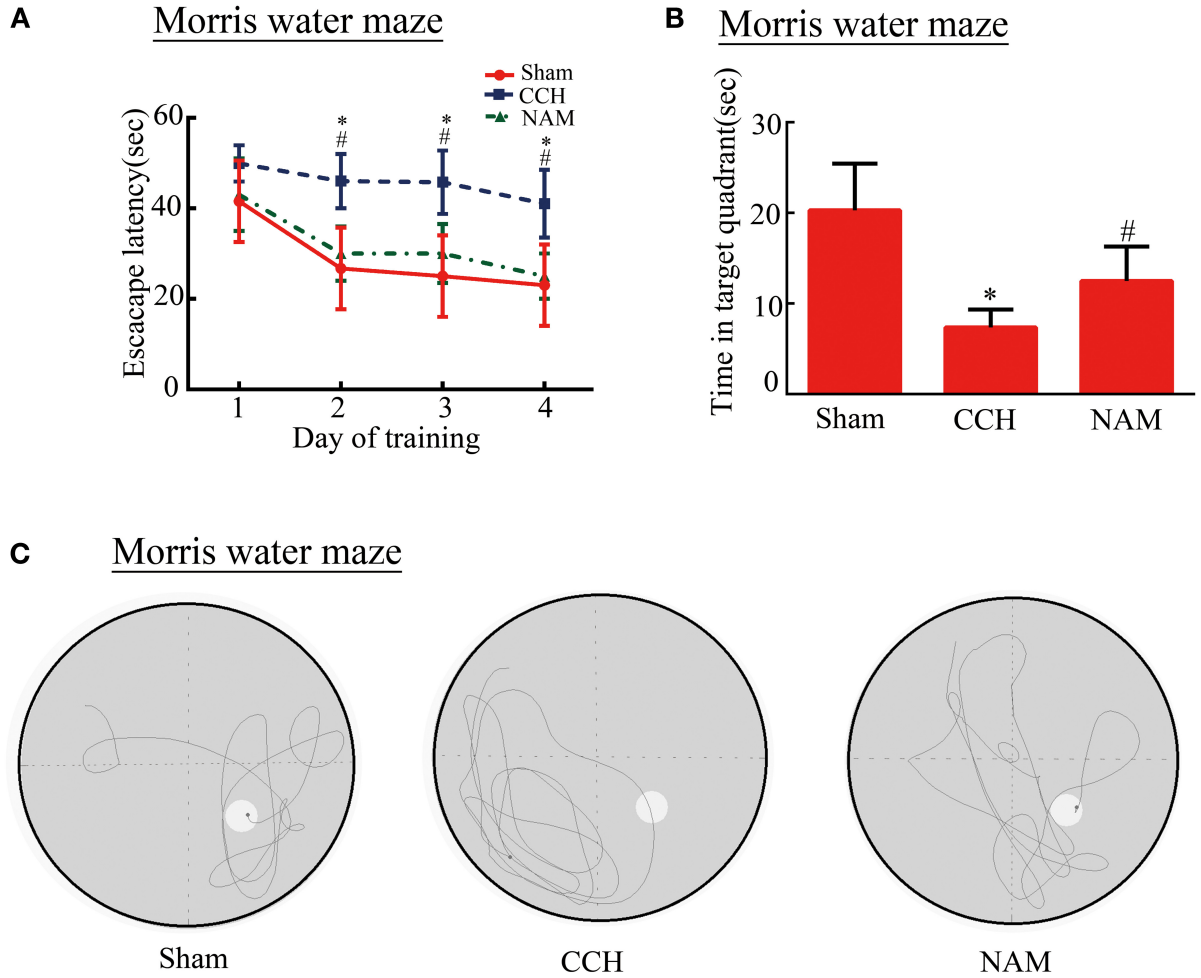
Effect of NAM on mouse cognitive function (*n* = 8/group). **(A)**: Escape latency of mice; **(B)**: Time spent in target quadrant; and **(C)**: The recorded swim trajectories of three groups in probe trial of MWMT. *p < 0.05, compared with sham group; ^#^*p* < 0.05, compared with CCH group.

The spatial reference learning and memory were examined using the Morris water maze test. Our results show that the ability of CCH mice to find an invisible platform is impaired compared to mice in the sham group in the acquisition trial. However, NAM treatment partially reversed the cognition impairment in CCH mice (Figures 2A–C). The data revealed that escape latency was significantly increased in CCH mice compared to the sham group. And NAM treatment suppressed the escape latency increase in CCH mice (*p* < 0.05; Figure 2A). The time spent in the target quadrant was significantly lower in the CCH group than in the sham group, but they were significantly increased by NAM (*p* < 0.05; Figure 2B). These results suggest that hypoperfusion could lead a tendency of impairment in spatial reference learning and memory. Meanwhile, NAM could significantly alleviate this impairment.

### Effect of NAM on Anxiety and Depression-Like Behaviors in CCH Mice

**Figure 3:**
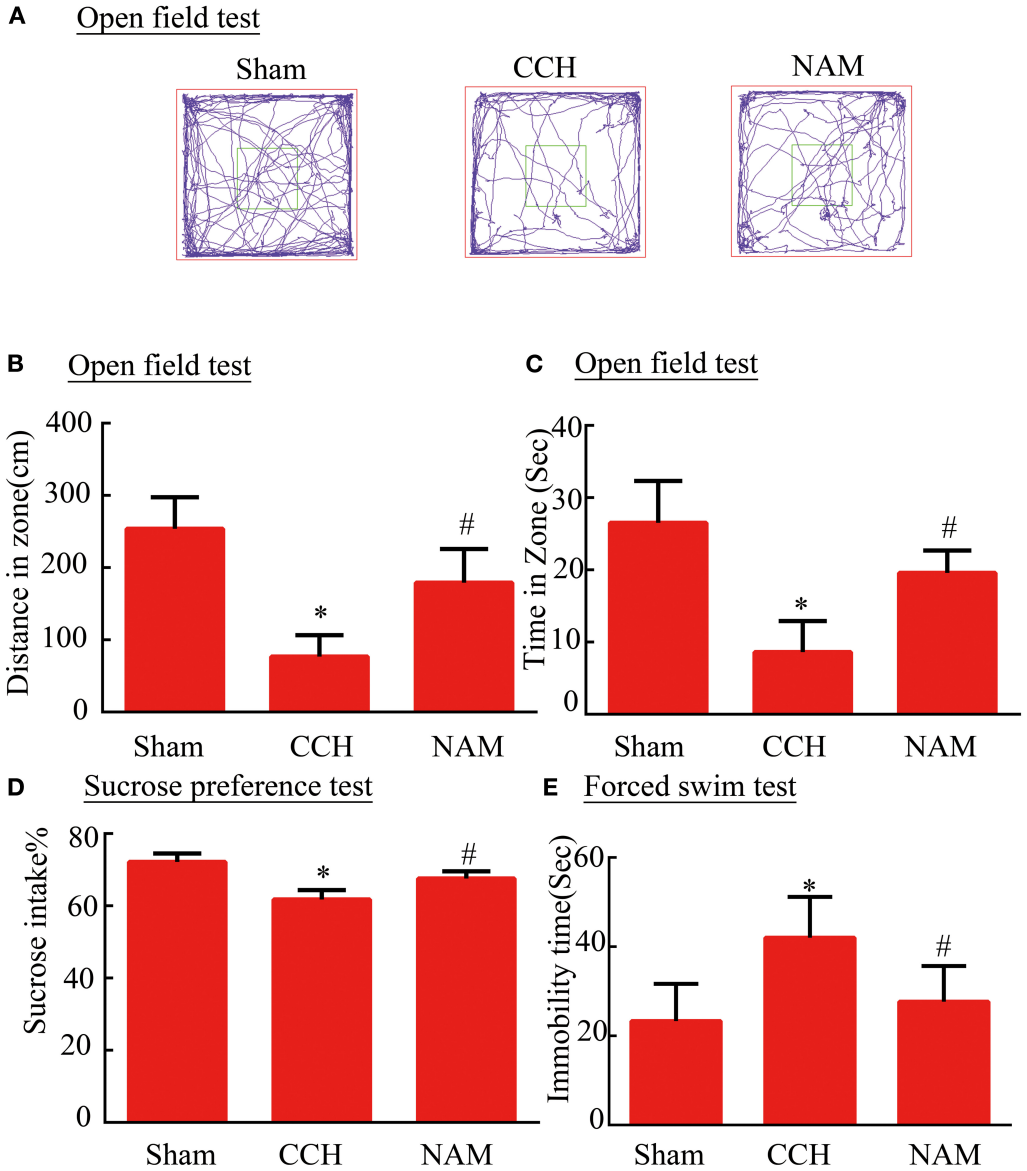
Effect of NAM on behavioral performance (*n* = 8/group). **(A)**: Typical movement tracks, as shown by trace lines in the OFT; **(B)**: Total distance traveled in the central zone; **(C)**: Time spent in the central region; **(D)**: Mice performance in SPT; and **(E)**: Immobility time of mice in FST. *p < 0.05, compared with sham group; #*p* < 0.05, compared with CCH group.

To test whether NAM contributes to anxiety and depression-like behaviors in CCH mice, the open filed test (OPT), SPT, and FST were performed (Figures 3A–E). After BCAS, total distance traveled and time spent in the central zone were decreased significantly in the OPT in the control group, while NAM treatment significantly elevated the distance and spent less time in the central zone compare to the control group (*p* < 0.05; Figures 3B,C). In SPT, CCH induced a significant reduction of anhedonia in the control group than in the sham group, but it was significantly increased by NAM (*p* < 0.05; Figure 3D). The results showed that mice in the control group spent more time of immobility, when compared with the sham group (*p* < 0.05; Figure 3E). The NAM treatment significantly decreased the hopelessness induced by CCH (*p* < 0.05; Figure 3E). These results showed NAM treatment could decrease the anxiety and depression-like behaviors caused by CCH.

### Effect of NAM on Histological Changes in White Matter

**Figure 4:**
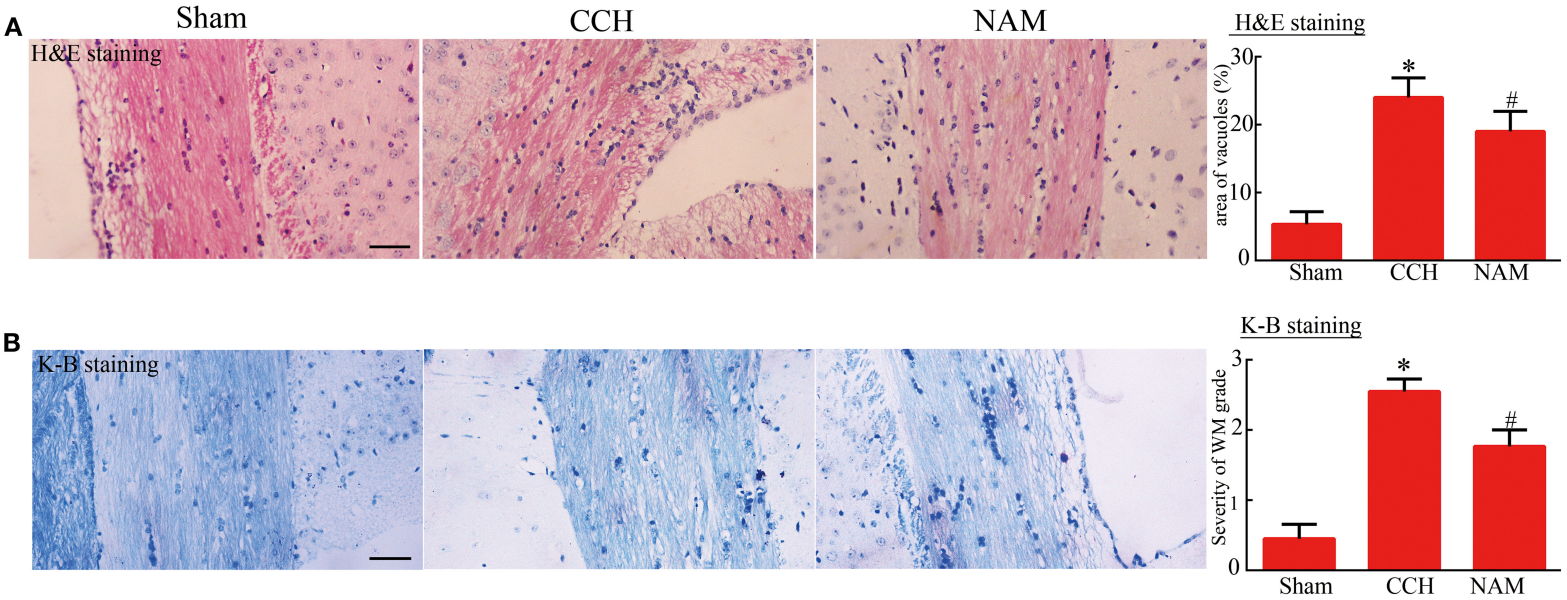
Effect of NAM on histological changes in white matter (*n* = 4/group). **(A)**: Vacuole area as determined via H&E staining (scale bar = 100 μm); and **(B)**: Degree of white matter damage as revealed by Kluver-Barrera staining (scale bar = 100 μm). *p < 0.05, compared with sham group; ^#^*p* < 0.05, compared with CCH group.

As shown in Figure 4, vacuole-like changes in the corpus callosum of CCH mice were significantly increased, relative to the sham group, but they were significantly reduced by NAM treatment (*p* < 0.05; Figure 4A). Similarly, histologic score (index of myelin injury) was significantly higher in the CCH group than in the sham group, but it was significantly reduced by NAM (*p* < 0.05; Figure 4B). The data indicated the protection of NAM for white matter damage in CCH.

### Effect of NAM on White Matter Remodeling and Synaptic Plasticity

**Figure 5:**
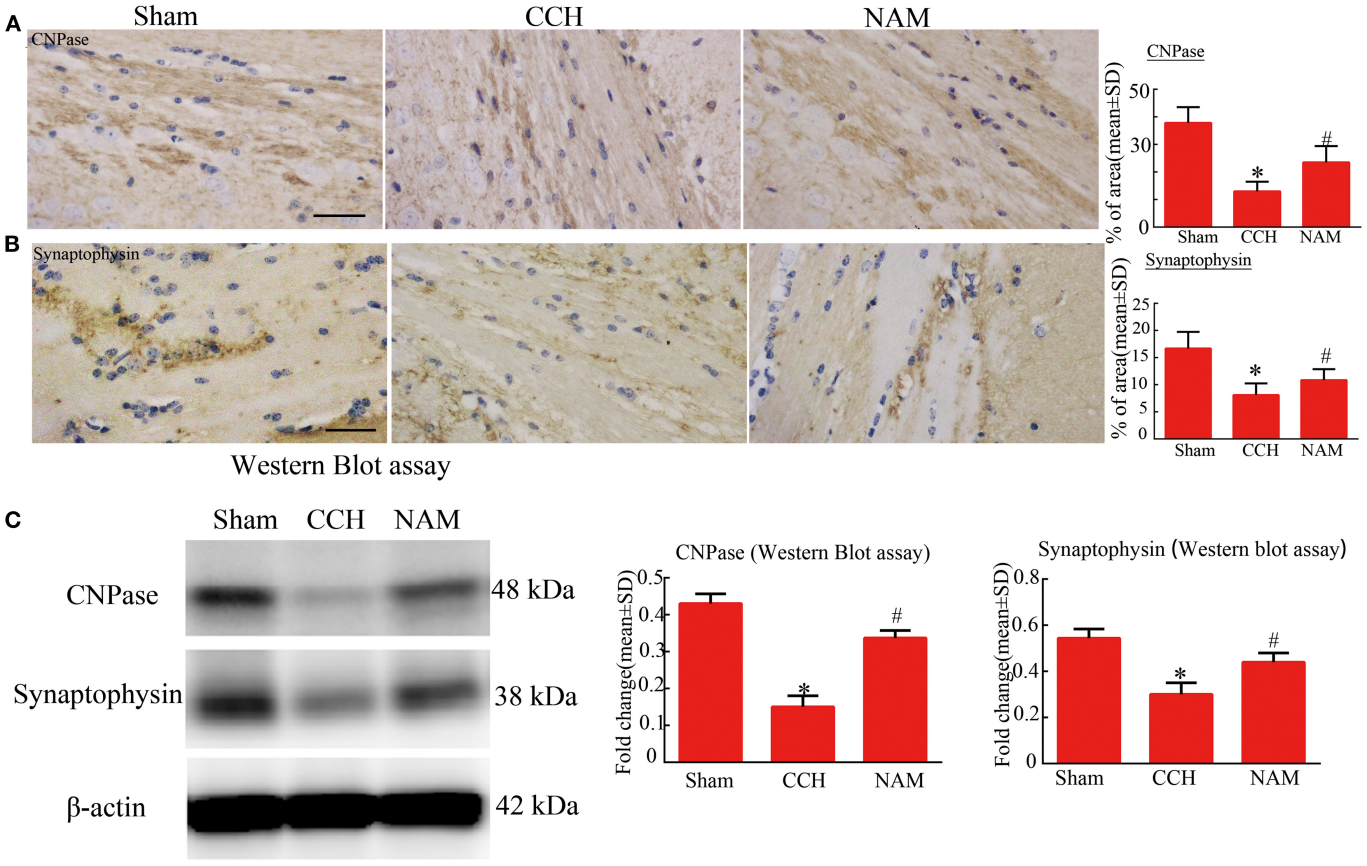
Effect of NAM on white matter remodeling and synaptic plasticity (*n* = 4/group). **(A)**: Expression of CNPase as measured via immunohistochemical staining (scale bar = 50 μm); **(B)**: Expression of synaptophysin as measured using immunohistochemical staining (scale bar = 50 μm); and **(C)**: Levels of expression of CNPase and synaptophysin proteins as measured via Western blotting. *p < 0.05, compared with sham group; ^#^*p* < 0.05, compared with CCH group.

The results of immunohistochemical staining and Western blotting showed that the protein expressions of CNPase and synaptophysin were significantly downregulated

in the CCH group, relative to the sham group, but they were significantly upregulated by NAM (*p* < 0.05; Figures 5A–C). The results revealed the myelin remodeling and synaptic plasticity in CCH-induced white matter injury.

## Discussion

Chronic cerebral hypoperfusion (CCH)-induced changes in subcortical white matter appear as bright areas of high signal intensity in diffusion tensor magnetic resonance imaging (DT MRI) (25). The risk factors associated with these lesions include old age, history of ischemic cerebrovascular disease, and hypertension. There is a high incidence of subcortical MRI lesions in patients with Alzheimer's disease and senile dementia of the Alzheimer type (SDAT) regardless of their vascular risk factors. Chronic cerebral hypoperfusion (CCH) promotes intracellular and extracellular amyloid-β (Aβ) peptide deposition via upregulation of the expression of β-secretase 1 (26). It is a major contributor to vascular cognitive impairment and dementia (VCID). Chronic cerebral hypoperfusion (CCH) is characterized by arteriolar occlusion, lacunes and white matter changes (27). Damage to white matter retards memory and slows down decision making process (28). It has been reported that cognitive domain (spatial reference memory) is significantly reduced in CCH mice (29). Moreover, it is now recognized that there is considerable overlap between VCID and Alzheimer's disease. The present study investigated the effect of NAM on CCH-induced cognitive impairment and white matter damage in mice. The results showed mild deficits in cognition in CCH mice manifested as memory impairment, depression, and anxiety-like behaviors. However, NAM treatment significantly reversed these deficits.

The hallmarks of CCH-induced cognitive deficits include white matter damage, blood-brain barrier (BBB) damage, and endothelial dysfunction (30, 31). however, white matter injury is the most prominent of all the pathological changes due to CCH (32). Cognition has been linked to brain frontal subcortical circuits and periventricular white matter junction (33). In this study, histopathological examination of brain tissues of CCH mice revealed decreased myelin density and extensive vacuole-like changes in the corpus callosum adjacent to the lateral ventricle. However, NAM significantly improved the histology of mice brain tissues. The corpus callosum consists of white matter supplied with arterial blood from branches of the internal carotid artery and middle cerebral artery that are not immediately or entirely compensated by redistribution of collateral circulation (34). The accentuated vulnerability of the corpus callosum in ischemia-related white matter damage has been implicated in cognitive impairment and imbalance (35, 36). Clinically, increased white matter grades measured using Fazekas's grading scale are positively correlated with cognitive decline (37). The results of this study are in agreement with those of previous studies (35, 36).

Myelin remodeling and synaptic plasticity are involved in white matter injury (38). Oligodendrocytes (OLs) are a type of large glial cells found in the central nervous system. They produce the myelin sheath that insulate neuronal axons. Thus, damage to OLs reduces myelin synthesis. Synaptic plasticity enhances axonal and dendritic regeneration as well as reorganization within cortical motor areas (39). White matter damage (defined as axonal degeneration and secondary demyelination) impairs nerve impulse conduction and evokes serious neurological dysfunction. Successful regeneration of OLs and synaptophysin are essential for remyelination and axonal preservation after brain injury. Axonal reorganization via sprouting of nearby axons has been implicated in spontaneous recovery after infarction in rodent models of acute stroke (40).

In this study, the results of immunohistochemical staining and Western blotting showed that the protein expressions of CNPase and synaptophysin were significantly downregulated in CCH group, relative to sham group, but they were significantly upregulated by NAM. Nicotinamide (NAM) is vital for the production and maintenance of mature neurons (41). It is used for the treatment of neurodegenerative diseases, neurotrauma, peripheral nervous disease and acute stroke. Studies have focused on the mitigating effects of NAM on behavioral impairment, cellular degeneration and remyelination in ischemic brain injury (17, 42, 43). The results of this study suggest that NAM may promote white matter remyelination and neurological regeneration in CCH-induced white matter injury.

The ischemia phase of CCH is persistent and mild, when compared with acute stroke (44). The results of previous studies showed that cognitive deficits occurred as early as 2 weeks after hypoperfusion, and the severity of the resultant impairment was proportional to the duration of hypoperfusion (5). Brain injury caused by CCH might related to microvascular dysfunction introduced by metabolic syndrome (45). Long-standing reduction in oxygen and energy supply might lead to oxidative stress and neuroinflammation related to cognitive impairment in neurodegeneration. The oxidative stress and neuroinflammation could explain extra-neuronal accumulation of β-amyloid peptide (Aβ) found in the senile plaques of hippocampal CA1 region introduced by CCH (46). Endoplasmic reticulum stress plays a pivotal role in misfolded proteins' accumulation in the ER lumen in CCH. Increased misfolded proteins could accelerate chronic inflammation and cell apoptosis (45). Moreover, the degree of white matter damage was also associated with the period of hypoperfusion. (23). Blood-brain barrier damage has been implicated in the pathogenesis of white matter lesions in CCH. The damage could occur to a small degree at 3 h, and markedly on day 3 in the paramedian part of the corpus callosum. The damage of blood-brain barrier was earlier than that of white matter fiber bundle. That indicated the early injury mainly concentrated in endothelial cells, pericytes and collagen fibers (47). With the prolongation of hypoperfusion time, white matter fiber damage gradually appeared and aggravated.

## Limitations of the Study

The study has some limitations. The mechanism of action of NAM was not investigated. Mechanisms for neuroprotection by nicotinamide might include maintain DNA stability, preventing neuron, trophic nerve, phagocytosis, and anti-inflammation. Blood-brain barrier injury, angiogenesis and neuronal apoptosis may be involved. Further research is needed. Moreover, the duration of treatment was short. In addition, the likely long-term toxic/biochemical effects of NAM were not studied.

## Conclusion

The results of this study indicated that NAM could reduce the damage of white matter caused by hypoperfusion, increase the myelin remodeling and synaptic plasticity, and improve cognitive function in mice with CCH.

## Data Availability Statement

The raw data supporting the conclusions of this article will be made available by the authors, without undue reservation.

## Ethics Statement

The animal study was reviewed and approved by Institutional Animal Care and Use Committee of Nanjing Medical University.

## Author Contributions

BL and JS: perception and design, operation, and final approval of the version to be published. GZ and LJ: participation in the whole work, drafting of the article, and data analysis. All authors contributed to the article and approved the submitted version.

## Conflict of Interest

The authors declare that the research was conducted in the absence of any commercial or financial relationships that could be construed as a potential conflict of interest.
